# A Retrospective Comparison of Aeroallergen Sensitization Among Different Allergic Diseases in Guangzhou, China

**DOI:** 10.1155/mi/8896646

**Published:** 2024-12-31

**Authors:** Chao Yang, Qingxiang Zeng, Gen Lu, Haipian Li, Wenlong Liu

**Affiliations:** ^1^Department of Otolaryngology, Guangzhou Women and Children's Medical Center, Guangzhou Medical University, Guangzhou 510623, China; ^2^Department of Respiratory, Guangzhou Women and Children's Medical Center, Guangzhou Medical University, Guangzhou 510623, China; ^3^Department of Dermatology, Guangzhou Women and Children's Medical Center, Guangzhou Medical University, Guangzhou 510623, China

**Keywords:** aeroallergen sensitization, allergic rhinitis, asthma, atopic dermatitis, children, eczema, urticaria

## Abstract

**Objective:** Numerous studies have reported on the types of aeroallergen sensitization in various pediatric allergic diseases, but limited data compared the types of aeroallergen sensitization across different pediatric allergic diseases. The aim of this study is to explore the nature and significance of aeroallergen sensitization in diverse pediatric allergic conditions.

**Methods:** A comparative analysis was carried out on aeroallergen sensitization in children suffering from allergic diseases who visited the Otolaryngology, Respiratory, and Dermatology Departments between January 2019 and December 2023. The evaluation of the specific immunoglobulin E (IgE) response to various inhalant allergens was done using the ImmunoCAP 100 system.

**Results:** Mites remain the main aeroallergen for skin and respiratory allergic diseases, especially respiratory diseases. Dog dander, grass pollen, and mold are more common in skin allergic diseases. The differences in dog dander and grass pollen among the three groups are more pronounced in children aged 1–6, while the differences in fungi are more pronounced in children aged 7 years and above. Seasonal changes have a greater impact on the sensitization rates of cockroaches, grass pollen, and molds.

**Conclusions:** Our results demonstrate the distribution and differences of allergen types among common pediatric allergic diseases, providing a theoretical basis for preventing the development of different allergic diseases and avoiding aeroallergens.

## 1. Introduction

Over the past 30 years, allergic conditions such as asthma, rhinitis, and atopic dermatitis (AD) have been increasing worldwide, particularly among children [1, 2]. Children suffering from these allergies often experience a decline in their quality of life and increased healthcare utilization [3, 4]. Asthma and rhinitis typically manifest later in childhood, whereas AD often starts early in infancy and often remits until puberty [5]. Allergic diseases often manifest concurrently, displaying varying degrees of severity and a sequential pattern of development, highlighting the importance of genetic predisposition, complex processes, and heredity in these conditions [6–8].

Exposure to aeroallergens follows a complex pattern influenced by both individual and global factors like pollution, climate, heredity, and birth circumstances [9]. Indoor and outdoor aeroallergens are the two primary categories of allergens. Indoor aeroallergens encompass mold, cockroaches, animal dander or fur, and house dust mites (HDMs), whereas outdoor aeroallergens primarily include pollens from grass, trees, and weeds [9].

While numerous studies have reported on the types of aeroallergen sensitization in various pediatric allergic diseases, there is limited data comparing the types of aeroallergen sensitization across different pediatric allergic diseases. The aim of this study is to explore the nature and significance of aeroallergen sensitization in diverse pediatric allergic conditions.

## 2. Methods

### 2.1. Study Population

A retrospective analysis was conducted on the data collected from individuals diagnosed with allergic conditions (specifically allergic rhinitis (AR), allergic asthma (AS), and AD) at our hospital from Department of Otolaryngology, Department of Respiratory, and Department of Dermatology, spanning from January 2019 to December 2023. Information related to demographics, including age, gender, atopic diseases, and the results of serum immunoglobulin E tests for common airborne allergens, was gathered. Written informed consent was obtained from the parents, and the study adhered to the approval granted by the local ethics committee.

### 2.2. Determination of Serum Total and Specific Immunoglobulin E (IgE) Levels

The atopic status of individuals was evaluated by determining the levels of serum IgE (using equipment from Phadia, Uppsala, Sweden), which were specific to commonly inhaled allergens such as dust mites, grass pollens, pets, molds, and cockroaches. The types and methods of aeroallergen detection adopted by different departments are unified.

### 2.3. Diagnosis of Allergic Diseases

The diagnosis of allergic disorders was determined through the utilization of the revised ISAAC questionnaire [10]. Subjects were categorized as cases if their parents responded affirmatively to inquiries such as “Has a doctor ever diagnosed you with AS?,” “Has a doctor ever diagnosed you with AR?,” or “Has a doctor ever diagnosed you with AD?”

### 2.4. Statistical Analysis

Statistical analysis was performed utilizing the SPSS software version 22. To compare proportions among various groups, the *χ*^2^ test was utilized. For continuous data, the Mann–Whitney *U* test was applied for comparison purposes. A significance level of *p*  < 0.05 was set as the criterion for determining statistical significance.

## 3. Results

### 3.1. Comparison of Type of Aeroallergen Sensitization of Children With Different Allergic Diseases

A total of 557,213 and 3828 children with sensitization to aeroallergens with different allergic diseases were included in the study. Analysis revealed no significant differences among the groups in terms of average age, sex ratio, monosensitized ratio, or polysensitized ratio (Table 1; *p* > 0.05).

The sensitization rates to *D. farinae*, *D. pteronyssinus*, dog dander, and grass pollen in AD were significantly different from AR and AS (Table 2; *p*  < 0.05). The sensitization rate to molds was significantly different between AD and AR (Table 2; *p*  < 0.05). The sensitization rates to cockroaches and cat dander had no significant difference among groups (Table 2).

### 3.2. Comparison of Type of Aeroallergen Sensitization of Children With Different Allergic Diseases According to Age

In preschool children (1–3 years old), the positive rates of sensitization to *D. farinae*, *D. pteronyssinus*, dog dander, and grass pollen in AR had significant difference compared with AD (Table 3).

In school-age group, the positive rates of sensitization to *D. farinae*, dog dander, grass pollen, and molds in AR had significant difference compared with AD (Table 3). Moreover, the positive rates of sensitization to mold in AS had significant difference compared with AD.

In youngsters group, the positive rates of sensitization to *D. farinae*, *D. pteronyssinus*, cat dander, and molds in AR had significant difference compared with AD (Table 3).

### 3.3. Comparison of Type of Aeroallergen Sensitization of Children With Different Allergic Diseases According to Gender

In male group, the sensitization rates to *D. farinae*, *D. pteronyssinus*, dog dander, grass pollen, and molds in AD were significantly different from AR (Table 4; *p*  < 0.05). Moreover, the positive rates of sensitization to *D. pteronyssinus* in AS had significant difference compared with AD. In female group, the sensitization rates to *D. farinae*, *D. pteronyssinus*, dog dander, and grass pollen in AD were significantly different from AR (Table 4; *p*  < 0.05).

### 3.4. Comparison of Type of Aeroallergen Sensitization of Children With Different Allergic Diseases According to Seasons

In spring, the sensitization rates to *D. farinae*, *D. pteronyssinus*, grass pollen, and molds in children with AD were significantly different with those with AR (Table 5; *p*  < 0.05).

In summer, the sensitization rates to *D. farinae*, *D. pteronyssinus*, dog dander, and molds in children with AD were significantly different with those with AR (Table 5; *p*  < 0.05).

In autumn, the sensitization rates to *D. farinae*, *D. pteronyssinus*, and dog dander in children with AD were significantly different with those with AR (Table 5; *p*  < 0.05). The sensitization rates to cockroach in children with AR was significantly different with those with AS and AD (Table 5; *p*  < 0.05).

In winter, only the positive rate of sensitization to *D. farina* and *D. pteronyssinus* in children with AD were significantly different with those with AR (Table 5; *p*  < 0.05).

## 4. Discussion

In this study, we compared the types of aeroallergen sensitization among three different department from the same children's hospital. Our research shows that the types of aeroallergens in different allergic diseases are not the same. In respiratory (including upper and lower respiratory) allergic diseases, the sensitization rates to mite are significantly higher than that of children with skin allergic diseases, which is consistent with previous studies. In 2009, Li et al. [11] surveyed 6304 asthmatics and/or rhinitis sufferers spread across 17 cities in four regions of China. Their findings revealed that *D. farina* and *D. pteronyssinus* were the predominant aeroallergens in individuals affected by AR. It is worth mentioning that sensitization to HDMs was most prevalent in China's southwestern region. Moreover, mite sensitization is associated with the risk of asthma in children and adults as described by several studies [12–16]. Skin allergic reactions typically require direct skin contact, and as the body's first line of defense, the skin's barrier function may reduce the penetration of allergens to some extent. In contrast, the respiratory tract has a weaker barrier function and is more susceptible to mite irritation. In terms of structure, the airway epithelium possesses intermediate characteristics between the epidermis and the intestinal epithelium, meaning it is more permeable than the epidermis, but less permeable than the intestinal epithelium [17]. Interestingly, the sensitization rates of dog dander, mold, and grass pollen are significantly higher in skin diseases than in respiratory diseases. This may be related to the small size of these allergens (micrometer scale, compared to millimeters for mites), which makes it easier for them to penetrate the skin barrier.

When grouped by age, we found that only the sensitization rate of mites in AR was significantly higher than that in AD across different age subgroups, suggesting that mite sensitization has the greatest impact on the upper respiratory tract. The sensitization rates of dog dander, molds, and grass pollen did not show a consistent trend across age groups, possibly due to the small sample size. Among molds-sensitized allergic diseases, we found that the sensitization rate of fungi increased with age in all three allergic diseases. When patients were grouped by gender, we found that the sensitization rates of various aeroallergens were consistent with the overall trend, indicating that gender has little effect on sensitization.

We also observed differences in sensitization rates of various aeroallergens across seasons. As an indoor allergen, the sensitization rate of mites remains relatively consistent across seasons. There was no significant difference in the sensitization rate of cockroaches among the three allergic diseases, but sensitization to the respiratory tract was significantly higher in autumn, possibly related to increased cockroach reproduction during early autumn. AD caused by grass pollen and molds significantly increase during spring and summer, possibly related to pollen dispersal and seasonal humidity.

Previous studies had shown that pediatric AD patients display the highest positivity for HDM in atopy patch tests (APTs), accounting for 48.9%. Moreover, those with strong skin prick test (SPT) positivity towards HDM exhibit significantly worse AD severity [18]. Moreover, the severity of AD positively correlates with pollen levels [19].

Our study has some limitations. First, our sample size is limited. We recognize the limitations in statistical power resulting from the small sample sizes across different age and gender subgroups in our study. Despite these limitations, we have conducted rigorous statistical analyses to identify significant differences in aeroallergen sensitization rates among the three allergic diseases. While some differences did not reach statistical significance due to the small sample sizes, our findings still provide useful information on the trends and patterns of aeroallergen sensitization in pediatric allergic diseases. We plan to conduct future studies with larger sample sizes to further explore these trends and validate our findings. Second, our study was conducted at a single center, which may limit the generalizability of our findings to other populations. While our findings may not be directly applicable to all pediatric populations worldwide, they provide valuable insights into the distribution and differences of allergen types among three common allergic diseases in our specific patient cohort. Future multicenter studies with larger sample sizes are needed to further validate our findings and enhance their generalizability. Third, our allergen testing types are limited. Fourth, our retrospective study will reduce the consistency of the data. While retrospective studies inherently have limitations, we have taken steps to minimize biases and ensure the robustness of our findings. We used standardized diagnostic criteria and the ImmunoCAP 100 system to evaluate the specific IgE response to various inhalant allergens, which minimized variability in test results. We also conducted rigorous statistical analyses to identify significant differences in aeroallergen sensitization rates and ensured the accuracy and completeness of the data. For data cleaning, we purified and organized data by removing errors, duplicates, and incomplete information, thereby, ensuring the accuracy and consistency of the data.

Overall, our research results suggest that mites remain the main aeroallergen for skin and respiratory allergic diseases, especially respiratory diseases. Dog dander, grass pollen, and mold are more common in skin allergic diseases. The differences in dog dander and grass pollen among the three diseases are more pronounced in children aged 1–6 years, while the differences in fungi are more pronounced in children aged 7 years and above. Seasonal changes have a greater impact on the sensitization rates of cockroaches, grass pollen, and molds. Our results demonstrate the distribution and differences of allergen types among three common allergic diseases, providing a theoretical basis for preventing the development of different allergic diseases and avoiding aeroallergens.

## Figures and Tables

**Table 1:** Characteristics of allergens in children with different allergic diseases.

Characteristics	AD	AS	AR
Cases	557	213	3828
Age (years)	4.75 ± 3.02	6.66 ± 2.57	6.54 ± 2.85
Sex ratio (Male:female)	55%:45%	67%:33%	69%:31%
Monosensitized	13.5%	5.2%	5.1%
Polysensitized	86.5%	94.8%	94.9%
Total IgE (IU/mL)	176.2 (23.6–519.8)	215.4 (22.6–420.4)	234.7 (41.7–669.4)

Abbreviations: AD, atopic dermatitis; AR, allergic rhinitis; AS, allergic asthma; IgE, immunoglobulin E.

**Table 2:** Aeroallergen sensitization of children with different allergic diseases.

Aeroallergens	AD (%)	AS (%)	AR (%)	*p*
*D. farinae*	87.5	94.4^*∗*^	96.1^*∗*^	**0.0001**
*D. pteronyssinus*	87.1	93.5^*∗*^	95.0^*∗*^	**0.0001**
German cockroach	21.4	23.5	19.0	0.247
Cat dander	14.2	8.0	11.0	0.197
Dog dander	15.5	5.7^*∗*^	7.3^*∗*^	**0.0001**
Grass pollen	9.9	3.8^*∗*^	4.3^*∗*^	**0.0001**
Molds	13.7	9.0^*∗*^	6.3^*∗*^	**0.0001**

*Note:* Bold for *p* value with significant difference.

Abbreviations: AD, atopic dermatitis; AR, allergic rhinitis; AS, allergic asthma.

*⁣* ^*∗*^Compared with AD group, *p* < 0.05.

**Table 3:** Aeroallergen sensitization of children with different allergic diseases by age groups.

Aeroallergens	1–3 yrs			4–6 yrs			≥7 yrs		
	AD (%)	AS (%)	AR (%)	AD (%)	AS (%)	AR (%)	AD (%)	AS (%)	AR (%)
*D. farinae*	81.9	88.9	93.1^#^	90.4	94.6	95.9^#^	92.6	94.7	97.1^#^
*D. pteronyssinus*	82.4	77.8	93.3^#^	90.9	95.5	94.3	89.7	92.5	96.2^#^
German cockroach	16.0	22.3	10.1	21.0	19.9	15.8	31.2	28	24.7
Cat dander	10.7	0.1	6.4	14.3	10.0	8.8	20.0	6.5	14.5^#^
Dog dander	14.2	0.1	3.8^#^	16.4	5.5	5.6^#^	16.3	6.5	10.1
Grass pollen	12.4	11.2	2.2^#^	9.2	3.7	3.9^#^	8.2	3.3	5.2
Molds	9.8	0.1	4.4	16.4	4.6^#^	5.6^#^	16.3	15.1	7.7^#^

Abbreviations: AD, atopic dermatitis; AR, allergic rhinitis; AS, allergic asthma, yrs, years old.

^#^Compared with AD group, *p* < 0.05.

**Table 4:** Aeroallergen sensitization of children with different allergic diseases by gender.

Aeroallergens	Male			Female		
	AD (%)	AS (%)	AR (%)	AD (%)	AS (%)	AR (%)
*D. farinae*	86.0	93.8^#^	96.0^#^	89.3	95.8^#^	95.9^#^
*D. pteronyssinus*	85.7	93.1^#^	95.0^#^	88.9	94.3^#^	94.3^#^
German cockroach	24.9	25.2	20.8	17.2	20.0	15.8
Cat dander	12.8	9.1	11.0	16.0	5.8	8.8
Dog dander	15.1	7.0^#^	7.5^#^	16.0	2.9^#^	5.6^#^
Grass pollen	10.5	4.9^#^	4.9^#^	9.2	1.5^#^	3.9^#^
Molds	16.1	9.8	6.4^#^	10.8	7.2	5.6^#^

Abbreviations: AD, atopic dermatitis; AR, allergic rhinitis; AS, allergic asthma.

^#^Compared with AD group, *p* < 0.05.

**Table 5:** Aeroallergen sensitization of children with different allergic diseases by seasons.

Aeroallergens	Spring			Summer			Autumn			Winter		
	AD (%)	AS (%)	AR (%)	AD (%)	AS (%)	AR (%)	AD (%)	AS (%)	AR (%)	AD (%)	AS (%)	AR (%)
*D. farinae*	87.8	94.7	95.0^#^	88.4	92.7	97.0^#^	89.3	94.4	95.4^#^	86.9	97.3	96.6^#^
*D. pteronyssinus*	84.6	92.9	94.2^#^	89.4	94.2	96^#^	86.3	94.4	94.8^#^	86.9	91.7	94.3^#^
German cockroach	19.9	26.8	17.4	21.7	17.7	21.9	15.3	34.0^#^	19.3^*∗*^	21.3	13.9	14.6
Cat dander	14.6	12.5	11.2	14	8.9	10.7	7.6	5.7	9.6	15.6	2.8	13.2
Dog dander	10.2	9.0	7.4	16.4	4.5	7.7^#^	15.3	5.7	5.9^#^	12.3	2.8	8.2
Grass pollen	13.8	1.8	4.2^#^	7.7	4.5	4.7^#^	9.2	7.6	4.2	7.4	0.0	3.3
Molds	11.4	7.2	6.7^#^	15	10.3	7.1^#^	10.7	9.5	5.7	11.5	8.4	5.2

Abbreviations: AD, atopic dermatitis; AR, allergic rhinitis; AS, allergic asthma.

^#^Compared with AD group, *p* < 0.05.

*⁣* ^*∗*^Compared with AS group, *p* < 0.05.

## Data Availability

The data that support the findings of this study are available from the corresponding author upon reasonable request.
